# Effects of Three Different Bee Pollen on Digestion, Immunity, Antioxidant Capacity, and Gut Microbes in *Apis mellifera*

**DOI:** 10.3390/insects16050505

**Published:** 2025-05-08

**Authors:** Xin-Meng Li, Ying Wang, Li Lei, Ge Zhang, Bao-Hua Xu

**Affiliations:** Key Laboratory of Efficient Utilization of Non-Grain Feed Resources (Co-Construction by Ministry and Province), Ministry of Agriculture and Rural Affairs, Shandong Provincial Key Laboratory of Animal Nutrition and Efficient Feeding, College of Animal Science and Technology, Shandong Agricultural University, Tai’an 271018, China; 18211753152@163.com (X.-M.L.); wangying@sdau.edu.cn (Y.W.); leili_halcyon@163.com (L.L.); gezhang.bee@gmail.com (G.Z.)

**Keywords:** *Apis mellifera*, bee-collected pollen, nutrition, digestion, health, gut microbiota

## Abstract

The nutritional value of pollen for honeybees varies significantly depending on its source. This study examines the nutrient composition of three types of bee-collected pollen—Maize bee-collected pollen (MBP), Lotus bee-collected pollen (LBP), and Sunflower bee-collected pollen (SBP)—and their effects on honeybee digestion, immunity, and gut microbiota. Nutritional analysis revealed no significant differences in moisture, protein, and carbohydrate content among the three pollen types. However, sunflower bee-collected pollen exhibited a significantly lower crude fat content than maize bee-collected pollen and lotus bee-collected pollen, while lotus bee-collected pollen had a significantly higher crude ash content than the other pollen types. Additionally, notable differences in amino acid composition were observed across the three pollen types. Feeding assays demonstrated that honeybees consumed significantly more sunflower bee-collected pollen than maize bee-collected pollen or lotus bee-collected pollen. Honeybees fed sunflower bee-collected pollen exhibited reduced lipid deposition, enhanced immune enzyme activity, and increased expression of immune-related genes. Protein digestibility was highest in honeybees fed lotus bee-collected pollen, whereas protease and lipase activities were significantly lower in those consuming sunflower bee-collected pollen. Notably, honeybees fed sunflower bee-collected pollen had thinner midgut intestinal walls. Furthermore, the microbial structure of the honeybee gut was altered depending on the type of different pollen. In summary, honeybees had the worst digestibility of Sunflower bee-collected pollen; sunflower bee pollen had a greater effect on antioxidant and immune functions, and intestinal flora of honeybees. These findings underscore the influence of different types of pollen on honeybee nutrition, immunity, and gut microbiota, offering a foundation for optimizing pollen diets to support honeybee health.

## Simple Summary

Honeybees are ecologically important as pollinators. Bee-collected pollen is a natural food for honeybees, and the nutrient composition and nutritional effects on honeybees vary greatly among different pollens. Maize bee-collected pollen, lotus bee-collected pollen, and sunflower bee-collected pollen, as the main food sources of honeybees, are of great importance for their nutritional value, but research on these types of pollen is limited. In this study, we determined not only the nutrient composition of maize bee-collected pollen, lotus bee-collected pollen, and sunflower bee-collected pollen, but also their effects on digestion, immunity, and gut microbiology of honeybees. We found that the amino acid content of the three bee-collected pollen varied considerably, that honeybees digested sunflower bee-collected pollen the least, and that sunflower bee-collected pollen had a greater effect on antioxidant and immune functions, and intestinal flora of honeybees. This finding lays the foundation for rational selection of bee pollen diets.

## 1. Introduction

As one of nature’s most crucial pollinators, the honeybee (*Apis mellifera*) plays a pivotal role in both natural ecosystems and agricultural systems [1]. During pollination, honeybees acquire essential nutrients—pollen and nectar—that support their growth and development [2,3]. Pollen serves as the primary natural source of protein for honeybees, while also supplying lipids, vitamins, and minerals [4,5].

Depending on the plant species, growing conditions, and harvesting methods, pollen typically contains 14–30% protein, 1–10% lipids, 40–85% carbohydrates, and various microorganisms such as bacteria and fungi [6]. These variations in nutrient composition result in differing effects of pollen types on honeybee health [7]. The honeybee digestion efficiency also varies depending on the pollen type, with higher digestibility facilitating better nutrient absorption [8]. Studies have shown that pollen influences honeybee metabolism, immunity, and pathogen resistance [9]. At the same time, it reduces the sensitivity and resistance of individual honeybees to external threats [10]. Additionally, inadequate pollen availability can reduce honeybee populations by impairing their growth and resilience [11]. Northern China is a major production area for maize bee-collected pollen (MBP), lotus bee-collected pollen (LBP), and sunflower bee-collected pollen (SBP), and beekeepers often use these bee pollens as artificial supplemental feeding materials in the spring or during the period when outside sources of pollen are scarce. Although studies have linked pollen consumption to honeybee lifespan and immune function, knowledge of how different types of pollen affect gut microbiota and overall health remains limited.

The hindgut of honeybees is functionally analogous to the small intestine in mammals, hosting a dense community of microorganisms. These microbes primarily consist of a core microbiota and other associated microflora [12,13]. The rectal microbial community is predominantly composed of gram-positive lactobacilli species (*Lactobacillus nr. melliventris* and *Bombilactobacillus* spp.—formerly referred to as Firm-5 and Firm-4, respectively) along with various *Bifidobacterium* species [14,15]. These bacteria play a crucial role in digesting and metabolizing complex components of pollen, such as cellulose, pectin, and polysaccharides, which are otherwise difficult to digest and absorb [12,16,17]. For example, *Bifidobacterium* species possess glycoside hydrolase (GH) enzymes from different GH families, enabling them to break down various polysaccharides and pectins, thereby enhancing pollen digestion and nutrient absorption by the host [18,19]. Furthermore, the gut microbiota can influence the host’s immune system, potentially altering susceptibility to pathogens and parasites. *Frischella perrara*, for example, can stimulate melanin formation in the pyloric region of the gut, an innate immune response in insects that is often associated with tissue damage and pathogen invasion [20]. Concurrently, *Lactobacilli* and *Bifidobacteria* may provide protective benefits to the host by producing antimicrobial compounds that inhibit the growth of competing microorganisms [21]. Research has identified pollen as a critical source of gut microbes for honeybees [22,23]. Additionally, the composition of the honeybee gut microbiota can be regulated by pollen diets, which in turn impact honeybee health. Understanding the effects of pollen on gut microbiota is therefore essential for elucidating the relationship between pollen diversity and honeybee well-being.

To further elucidate the impact of the diversity in pollen sources on honeybee health, we determined the conventional nutrient composition of three bee-collected pollen commonly used in China, and explored their effects on the growth and development, digestion and absorption, immunity, and intestinal flora of honeybees. Through these experiments, we unveiled the underlying mechanisms by which different types of pollen may impact honeybee health.

## 2. Materials and Methods

### 2.1. Honeybee Selection

The honeybees (*Apis mellifera* L.) used in this study were obtained from the College of Animal Science and Technology at Shandong Agricultural University (Tai’an, China). A total of 750 newly emerged honeybees were obtained by constructing a sterile honeybee model [24], randomly divided into three groups, and fed pollen sterilized by UV irradiation as well as a sterile sugar-water solution (*n* = five cages per group, with 50 workers per cage). The honeybees were maintained in a sterile incubator (30 °C, 55% relative humidity; Wang et al., 2014) [25] and provided with three different pollen diets: maize bee-collected pollen (MBP), lotus bee-collected pollen (LBP), and sunflower bee-collected pollen (SBP). The diets consisted of a 10:1 weight ratio of pollen to water, and we took 500 g of each of the three types of pollen from the Experimental Station of Animal Husbandry Science and Technology of Shandong Agricultural University for the test. During the feeding period, each hive was supplied with a 50% sterile sucrose solution and water ad libitum. In order to determine the digestibility of pollen by honeybees, we subjected bees to 6 days of pollen feeding followed by 3 days of cessation of feeding. In order to determine the effect of pollen on immune function, antioxidant capacity, and intestinal microorganisms of honeybees, we fed pollen to honeybees for 9 days.

### 2.2. Determination of Nutrient Composition in Three Pollen Species

We took 50 g of each of the three types of pollen from the Experimental Station of Animal Husbandry Science and Technology of Shandong Agricultural University for the test. The nutrient composition (moisture, protein, fat, carbohydrate, and ash) of pollen was analyzed on a dry weight basis using AOAC procedures [26]. Moisture content was measured by drying samples at 60 °C for 48 h in a vacuum oven, cooling them in a desiccator, and weighing them until a constant weight was achieved. We equilibrated the pollen prior to all trials so that the pollen moisture content was at 6%. The sample protein content was determined using the Kjeldahl method (N × 6.25), while fat content was analyzed by extracting powdered samples with petroleum ether in a Soxhlet apparatus [27]. Ash content was assessed by incineration at 550 °C. Carbohydrate content was calculated by difference using the following formula: Carbohydrate = 100 − (g protein + g fat + g ash) [28]. Energy content was calculated as follows: Energy (kcal) = 4 × (g protein + g carbohydrate) + 9 × (g lipid) [29].

### 2.3. Determination of Amino Acid Content in Three Pollen Species

We took 50 g of each of the three types of pollen from the Experimental Station of Animal Husbandry Science and Technology of Shandong Agricultural University for the test. Amino acid content was analyzed through ultra-high-performance liquid chromatography (UPLC Waters, Milford, MA, USA). Separation was carried out on a Waters ACQUITY UPLC I-CLASS chromatograph equipped with a Waters UPLC HSS T3 column [2.1 mm (ID) × 150 mm (length), 1.7 µm (particle size)] maintained at 50 °C. The mobile phases consisted of 0.1% aqueous formic acid (phase A) and acetonitrile (phase B), with a flow rate of 0.5 mL/min and an injection volume of 5.0 µL (Supplementary Table S1). Mass spectrometry data were recorded using a Waters XEVO TQ-S Micro system (AB SCIEX, Boston, MA, USA) controlled by Masslynx Analysis software (Version 4.1, SCIEX, Boston, MA, USA).

### 2.4. Pollen Protein Digestibility

The diets consisted of a 10:1 weight ratio of pollen to water, and we took 500 g of each of the three types of pollen from the Experimental Station of Animal Husbandry Science and Technology of Shandong Agricultural University for the test. Protein digestibility was determined based on the method of Wang et al. (2014) [25]. A total of 600 newly emerged honeybee workers were collected and randomly divided into three groups of 150 individuals, each assigned to one of three feeding conditions: MBP, LBP, and SBP. An additional group of 150 workers was fed only a 50% sterile sucrose solution and water to assess endogenous protein levels in the midgut. Protein digestibility was calculated for each group of isolated workers by using a previously described formula [25]. For morphological analysis, midguts were extracted from three honeybees in three groups for light microscopy observation, and midgut thickness was measured following the method of Wang et al. (2014) [25].

### 2.5. Effect of Pollen on Enzyme Activity

We took six workers from each cage for enzyme activity measurements, and total protein concentration was measured using the MicroBCA Protein Assay Kit (CW2011S, CoWin Biosciences, Beijing, China). Enzyme activities included phenoloxidase (PO), lysozyme (Lys), Catalase (CAT), Superoxide Dismutase (SOD), as well as malondialdehyde (MDA) content and total antioxidant capacity. We took the midgut of 10 honeybees from each cage and used it to measure the protease and lipase activity in worker honeybees by using the enzyme-linked immunosorbent assay kit (MEIMIAN, Yancheng, China). Absorbance values were recorded using a UV-2000 ultraviolet spectrophotometer (Bio Tek Instrumen, Winooski, VY, USA), and enzyme activity was calculated based on the absorbance values obtained, normalized to the total protein concentration. Honeybee bodies, excluding the intestines, were dried and ground into a powder for analyzing protein and fat deposits.

### 2.6. Real-Time Quantitative Polymerase Chain Reaction Analysis

After 9 days of feeding, 10 honeybees from each group were rapidly frozen in liquid nitrogen and stored at −80 °C. Total RNA was extracted using a Total RNA Kit II kit (NCM Biotech, Suzhou, China), and RNA purity and concentration were determined using a micro UV-Vis spectrophotometer (DENOVIX, Wilmington, DE, USA). RNA was reverse-transcribed into cDNA using the Ackeri Evo M-MLV reverse transcription kit (Accurate Biology, Changsha, China) and stored in an ice box at −20 °C. Relative gene expression was assessed through real-time quantitative PCR, with β-actin as the internal reference gene. The primers were designed and synthesized by Biotech Bioengineering Co. (Shanghai, China). According to the TransStart Top Green qPCR SuperMix United States ABI7500 Kit (TransStart Top Green qPCR SuperMix United States ABI7500 Kit, ABI, Los Angeles, CA, USA), the PCR reaction mixture (20 μL total volume) included 10 μL of 2× SYBR^®^ Green Premix Pro Taq HS Premix (Rox Plus), 5 μL double-distilled water, 2 μL cDNA, and 1 μL primer. The thermal cycling conditions were as follows: (1) 30 s of predenaturation at 95 °C, (2) 40 amplification cycles (5 s of denaturation at 95 °C, and 30 s of extension at 60 °C), and (3) a single melting cycle from 65 °C to 95 °C. Relative gene expression levels were analyzed using CFX Manager software (version 1.1), and CT values were calculated using the 2(−ΔΔCT) method [30]. Primers for mitogen-activated protein kinase 3 (*MKP3*), glutathione S-transferase (*GstD1*), apolipoprotein D (*ApoD*), honeybee cytochrome (*Cyp4g11*), and *β-actin* genes are provided in Table 1.

### 2.7. Gut Microbial Analysis

For gut microbial analysis, rectums were extracted from 30 honeybees per group, collected into sterile centrifuge tubes for mixing, and immediately frozen in liquid nitrogen. The samples were sent to Guangzhou Chideo Biotechnology Co., Ltd. (Guangzhou, China). for high-throughput sequencing of the V3–V4 region of honeybee gut microorganisms. Library construction, computer sequencing, and sequencing data analysis were conducted by the same facility.

After genomic DNA was extracted, the V3–V4 region of the 16s rDNA gene was amplified using barcoded primers (341F: CCTACGGGNGGCWGCAG; 806R: GGACTACHVGGGTATCTAAT). Purified amplification products (i.e., amplifiers) were ligated with sequencing adapters, and a sequencing library was constructed for analysis using the Illumina platform (Illumina, San Diego, CA, USA).

Microbial diversity analyses included alpha diversity (within-sample diversity), beta diversity (between-sample diversity), species composition, and identification of indicator species. Raw reads were filtered to exclude low-quality reads and then assembled. The paired-end reads were merged into tags. The tags were filtered to obtain clean tags, which were further clustered to identify operational taxonomic units (OTUs). Chimera sequences were removed during clustering to ensure high-quality data for effective tags. OTU abundance was quantified based on effective tags, and intergroup differences were statistically tested.

### 2.8. Statistical Analysis

All statistical analyses were performed using SPSS (Version 21.0, SPSS Inc., Chicago, IL, USA) and GraphPad Prism (Version 9.0, GraphPad, La Jolla, CA, USA). For normally distributed data, one-way analysis of variance (ANOVA) with Tukey’s post-hoc test was used to compare groups. For non-normally distributed data, the Mann–Whitney U test was employed for pairwise comparisons, while the Kruskal–Wallis H test was used for multiple group comparisons. A *p* value of <0.05 was considered statistically significant. Error bars in figures represent the mean ± standard error of the mean (SEM), and different letters above bars indicate statistically significant differences.

## 3. Results

### 3.1. Determination of Nutrient Content of Pollen

The nutrient composition of the three pollen types (MBP, LBP, and SBP) were analyzed (Table 2). Protein, moisture, and carbohydrate contents showed no significant differences among the three types of pollen. Lipid content was significantly lower in SBP than in MBP and LBP. Ash content was significantly higher in LBP than in MBP and SBP. Energy content was significantly higher in MBP than in LBP and SBP.

### 3.2. Determination of Amino Acid Content of Pollen

All three pollen types contained the same amino acids, including 10 essential amino acids crucial for honeybee development (namely histidine, methionine, threonine, valine, tyrosine, lysine, isoleucine, leucine, phenylalanine, and arginine) and other nonessential amino acids (Table 3). Among the 10 essential amino acids, methionine content did not differ among the pollen types; histidine content was significantly higher in SBP than in MBP and LBP; valine, threonine, and tyrosine levels in MBP were not significantly different from those in LBP; lysine, isoleucine, and leucine contents in LBP were not significantly different from those in SBP; and phenylalanine and arginine contents were significantly higher in LBP than in MBP and SBP. For nonessential amino acids, no significant differences were noted in glycine and cystine contents among the three pollen types. Alanine and proline contents were significantly higher in MBP than in LBP and SBP. Aspartic acid and glutamic acid contents were significantly higher in LBP than in MBP and SBP.

### 3.3. Feed Intake, Nutrient Deposition, and Survival Rate of Honeybees Fed Different Pollen Types

**Figure 1:**
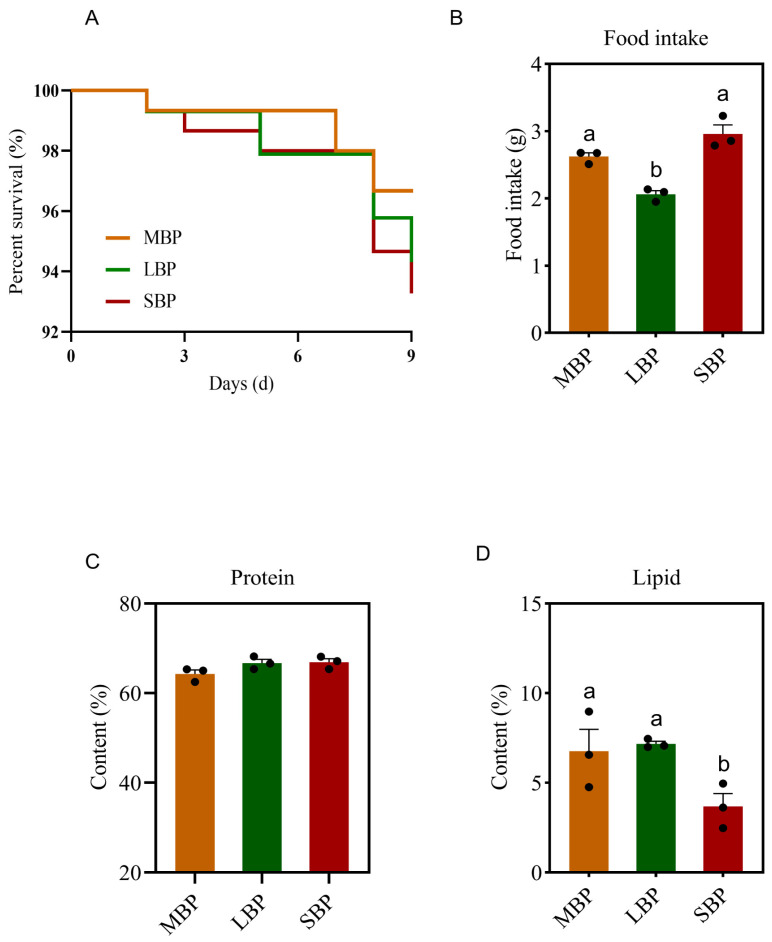
(**A**) Honeybee survival rate. (**B**) The intake of pollen during feeding. (**C**) Protein deposition in the honeybee body. (**D**) Lipid deposition in honeybee bodies. The date is shown as the mean ± SEM of three independent experiments (*n* = 3). Various letters above the bars indicate significant differences between two groups (*p* < 0.05) as determined by one-way ANOVA followed by Tukey’s multiple comparison test.

During the feeding period, the survival rate of honeybees was recorded daily. No significant differences in survival rates were observed among honeybees fed on the three pollen types, with all groups achieving a survival rate of more than 93% (Figure 1A).

We determined the amount of each pollen taken by the bees during the feeding period. We also recorded the amount of protein and lipid deposits in the bodies of honeybees consuming different pollen types. Honeybees fed SBP showed the highest daily feed intake, whereas those fed LBP consumed the least (Figure 1B). Although the amount of protein deposited in the honeybees’ bodies did not differ significantly among the three groups (Figure 1C), lipid deposition was significantly lower in bees consuming SBP than in those consuming MBP and LBP (Figure 1D).

### 3.4. Protein Digestibility and Digestive Enzyme Activity of Honeybees Fed Different Pollen Types

**Figure 2:**
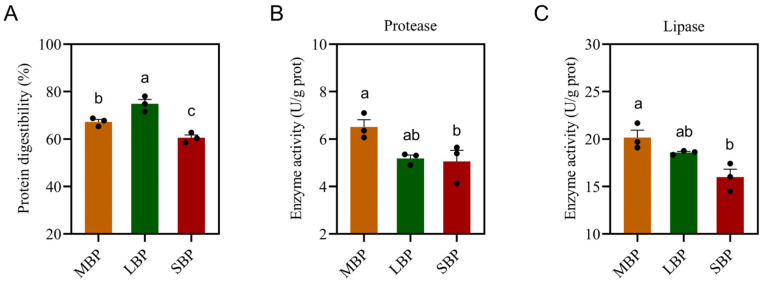
Digestibility of pollen protein and digestive enzyme activity in honeybees. (**A**) Protein digestibility. (**B**) Protease activity. (**C**) Lipase activity. Data are presented as mean ± SEM of three independent experiments (*n* = 3). Different letters above the bars indicate significant differences between two groups (*p* < 0.05), as determined by one-way ANOVA with Tukey’s multiple comparison test.

We measured protein digestibility and the activities of intestinal digestive enzymes, including protease and lipase (Figure 2). LBP had the highest protein digestibility, whereas SBP showed the lowest digestibility (Figure 2A). Protease (Figure 2B) and lipase (Figure 2C) activities were significantly higher in bees fed MBP and LBP than in those fed SBP. However, no notable differences in enzyme activities were observed between the MBP and LBP groups.

### 3.5. Effects of Different Pollens on Midgut Morphology in Honeybees

**Figure 3:**
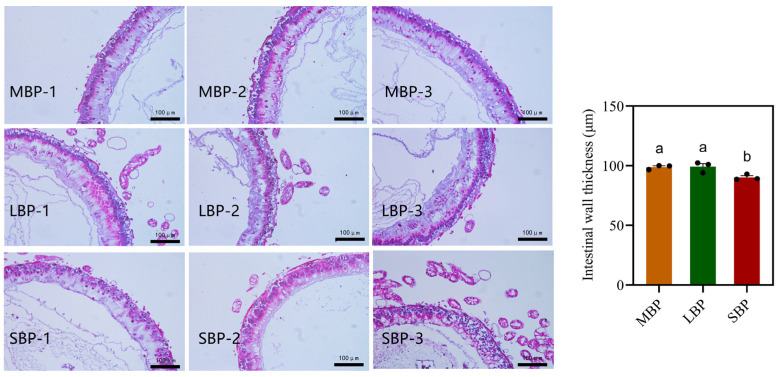
Light microscopy images of the midgut of *Apis. mellifera*. Intestinal wall thickness of midgut of honeybees fed CBP, LBP, and SBP. The scale is 100 μm. Data are presented as mean ± SEM of three independent experiments (*n* = 3). Different letters above the bars indicate significant differences between two groups (*p* < 0.05), as determined by one-way ANOVA with Tukey’s multiple comparison test.

Paraffin sections of the midgut were prepared, stained using hematoxylin and eosin, and examined under a light microscope. The thickness of the intestinal wall was measured. The midgut of honeybees fed SBP exhibited significantly thinner intestinal walls than those of honeybees fed CBP and LBP (Figure 3). This suggests that SBP may provide less support for gut structural development.

### 3.6. Effects of Different Pollens on Immune Enzyme Activities and Immune Gene Expression in Honeybees

**Figure 4:**
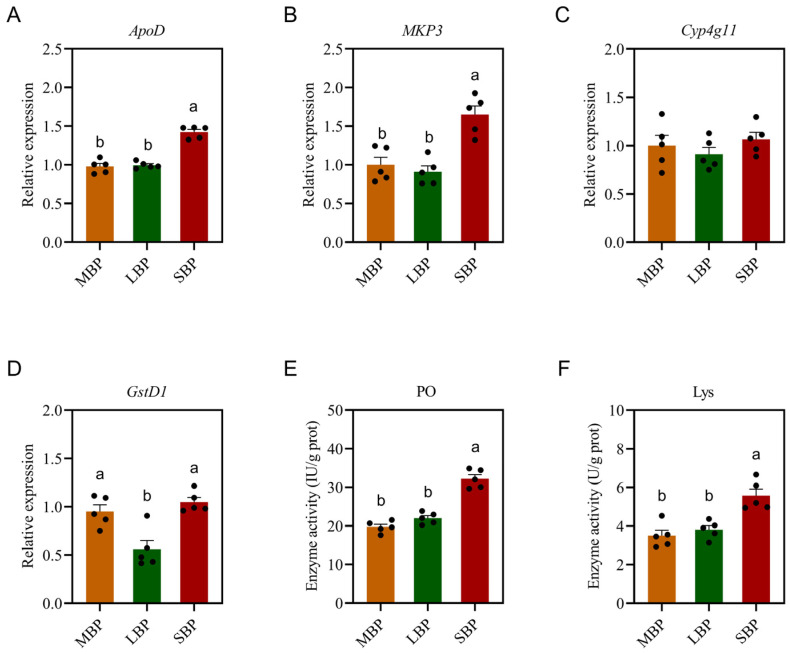
Effects of pollen on immune enzyme activities and immune gene expression in honeybees. (**A**) Relative expression of *ApoD*. (**B**) Relative expression of *MKP3*. (**C**) Relative expression of *Cyp4g11*. (**D**) Relative expression of *GstD1*. (**E**) PO activity. (**F**) Lys activity. Data are presented as mean ± SEM of three independent experiments (*n* = 5). Different letters above the bars indicate significant differences between two groups (*p* < 0.05), as determined by one-way ANOVA with Tukey’s multiple comparison test.

The impact of pollen diets on honeybee immunity was assessed by measuring the activities of immune enzymes and the expression of specific immune-related genes (Figure 4). Gene-expression analysis showed no significant difference in the expression of *ApoD* and *MKP3* in honeybees fed LBP compared to those fed MBP; however, both were lower than those fed SBP (Figure 4A,B). There was no significant difference in the expression of *Cyp4g11* among the three groups (Figure 4C). There was no significant difference in the expression of *GstD1* in honeybees fed SBP compared to those fed MBP, but both were significantly higher than those fed LBP (Figure 4D). Regarding enzyme activity, PO activity (Figure 4E) and Lys activity (Figure 4F) were significantly higher in honeybees fed SBP than in those fed LBP and MBP. These results suggest that SBP may enhance certain aspects of honeybee immunity, potentially as a response to dietary differences.

### 3.7. Effects of Different Pollens on Antioxidant Enzyme Activity in A. mellifera

**Figure 5:**
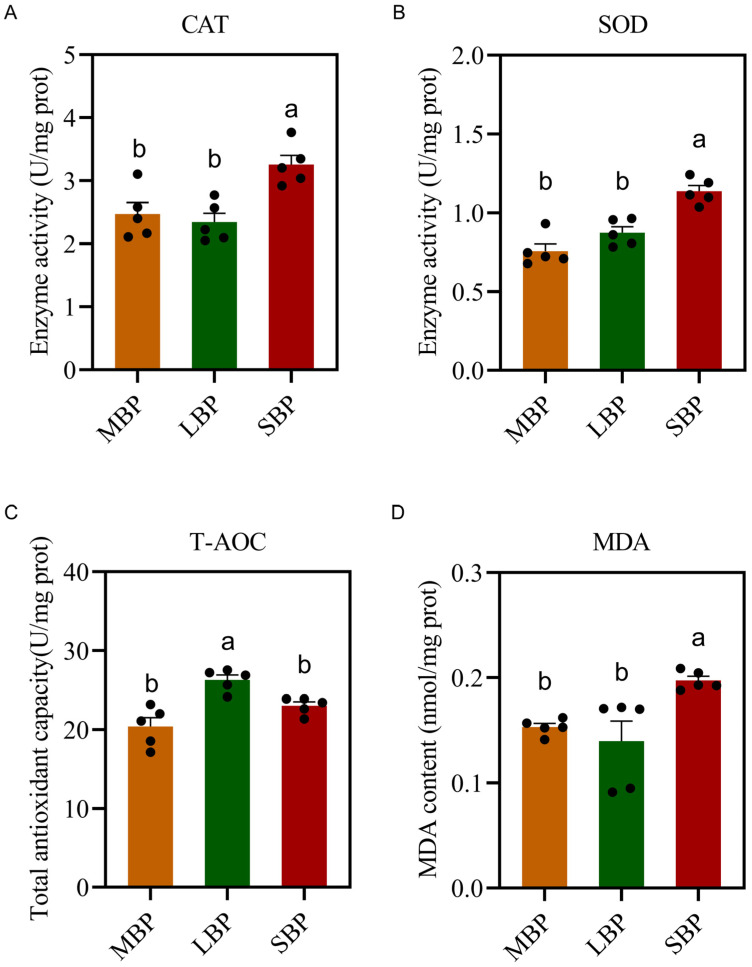
Effect of pollen on immunity enzyme activity antioxidant function in honeybees. (**A**) CAT activity. (**B**) SOD activity. (**C**) Total antioxidant capacity. (**D**) MDA content. Data are presented as means ± SEM of three independent experiments (*n* = 5). Different letters above the bars indicate significant differences (*p* < 0.05), whereas the same letters indicate that the difference is non-significant.

We measured the activities of antioxidant enzymes and overall antioxidant capacity following the ingestion of three types of bee pollen (Figure 5). The results revealed no significant differences in catalase (CAT) and superoxide dismutase (SOD) activities between honeybees fed LBP and MBP. However, honeybees fed SBP exhibited an increase in the activities of both enzymes (Figure 5A,B). Furthermore, the total antioxidant capacity of honeybees fed LBP and SBP was significantly higher (Figure 5C). By contrast, to evaluate the oxidative damage, we measured the malondialdehyde (MDA) content in the bodies of the honeybees, which was significantly higher in honeybees fed SBP than in those fed MBP and LBP (Figure 5D).

### 3.8. Effect of Pollen on Gut Microbial Composition

We analyzed the microbial composition in the hindgut of honeybees fed different types of pollen. The gut microbiota in each group was assessed through 16S rRNA sequencing from the fecal material of *A. mellifera*.

After initial data filtration, effective sequences (Supplementary Table S2) were obtained, and further clustering based on 100% identity was performed (Supplementary Table S3). Rarefaction curves indicated that the sequencing depth was sufficient to represent nearly all microbial diversity within each sample (Supplementary Figure S1), with a coverage greater than 99%.

**Figure 6:**
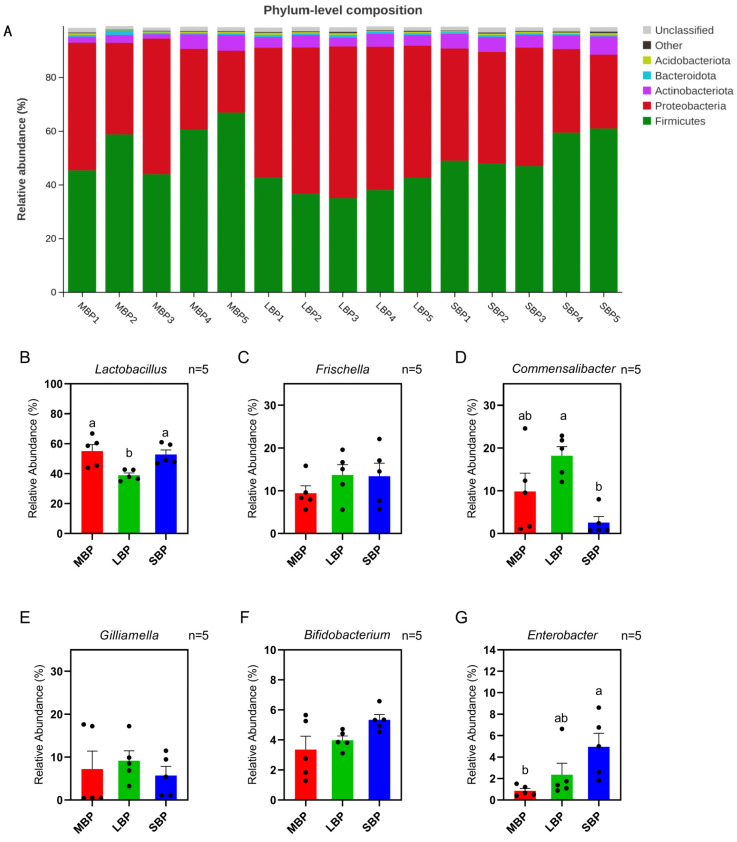
Comparison of major phyla and genera. (**A**) Relative abundances of the five most abundant bacterial phyla in all samples. (**B**–**G**) Comparison of the relative abundances of the genera. (**B**) *Lactobacillus*. (**C**) *Frischella*. (**D**) *Commensalibacter*. (**E**) *Gilliamella*. (**F**) *Bifidobacterium*. (**G**) *Enterobacter*. Data are presented as mean ± SEM (*n* = 5) of three independent experiments. Different letters above the bars indicate significant differences between two groups (*p* < 0.05), as determined by one-way ANOVA with Tukey’s multiple comparison test.

The average number of OTUs and the shared OTUs across different groups (MBP, LBP, and SBP) were visualized using a Venn diagram (Supplementary Figure S2). SBP had the highest number of OTUs, whereas LBP had the lowest. At the phylum level, LBP exhibited a low abundance of Firmicutes and a high abundance of Proteobacteria, whereas SBP had a high abundance of Actinobacteriota (Figure 6A; Supplementary Table S4). At the genus level, the relative abundances of *Frischella*, *Gilliamella*, and *Bifidobacterium* were similar across the three groups of honeybees fed different pollens. However, the relative abundance of *Lactobacillus* was lower in honeybees fed on LBP than in those fed on MBP and SBP, whereas the relative abundance of *Commensalibacter* was higher. Additionally, *Enterobacter* showed a higher relative abundance in honeybees fed SBP than in those fed the other two types of pollen (Figure 6B–G; Supplementary Table S5).

**Figure 7:**
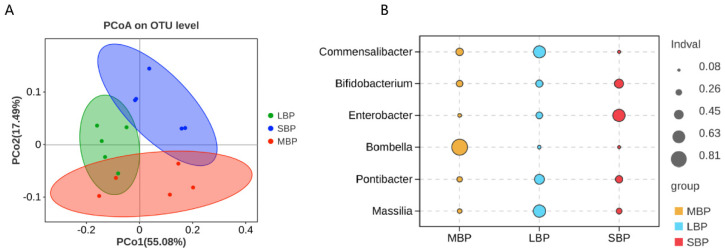
Principal coordinate analysis (PCoA) of Weighted–UniFrac distances and indicator analysis for all hindgut samples. (**A**) PCoA of each hindgut group. (**B**) Indicator analyses of each hindgut group.

We conducted an alpha diversity analysis using the Tukey HSD test (Supplementary Table S6). No significant differences in bacterial species richness were noted among bees consuming the three different pollens, as shown by the alpha diversity index (Supplementary Table S7). Regarding microbial community similarity at the genus level, principal coordinate analysis (PCoA) based on Weighted–Unifrac distances revealed that different pollen diets altered the microbial community composition in bee guts (Figure 7A; Supplementary Table S8). Indicator species analyses at the genus level highlighted distinct microbial signatures for each pollen type. Honeybees fed on MBP exhibited a high abundance of Bombella. In those fed on LBP, *Commensalibacter* and *Massilia* were predominant, whereas bees consuming SBP showed increased abundances of *Bifidobacterium* and *Enterobacter* (Figure 7B; Supplementary Table S9).

**Figure 8:**
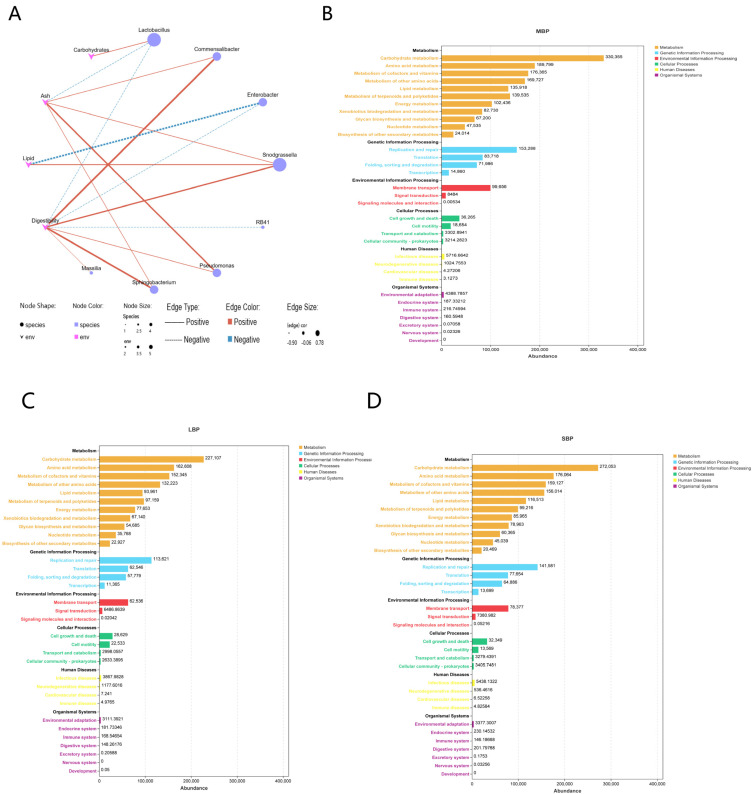
Co-occurrence network analysis and PICRUSt2−based microbial function analysis. (**A**) Pearson correlation analysis of microbial genera and nutritional components in the gut of honeybees consuming different types of pollen. (**B**–**D**) Microbial function in the hindgut of bees fed different types of pollen.

We analyzed the correlation between the nutritional components of pollen and the microbial genera in the honeybee gut (Figure 8A; Supplementary Table S10). The results revealed that carbohydrate content was positively correlated with *Lactobacillus*. Ash content showed a positive correlation with *Commentobacter*, *Snodgrassella*, *Pseudomonas*, and *Sphingomonas*, and a negative correlation with *Lactobacillus*. Lipid content was positively correlated with *Snodgrassella*, whereas negatively correlated with *Enterobacter*. Digestibility exhibited a positive correlation with *Commentobacter*, *Snodgrassella*, *Pseudomonas*, *Sphingomonas*, and *Massilia*, whereas a negative correlation with *Lactobacillus*, *Enterobacter*, and *RB41*.

We conducted a PICRUSt2−based predictive functional analysis. Sequencing data were categorized into six broad biological metabolic pathways: metabolism, genetic information processing, environmental information processing, cellular processes, human diseases, and organismal systems (Figure 8B–D; Supplementary Table S11). Among these, metabolism emerged as the dominant function. Further analysis of secondary functional levels of genes revealed 33 subfunctions, including carbohydrate metabolism, amino acid metabolism, lipid metabolism, and energy metabolism. Except for low-abundance functional genes related to signaling molecules and interactions, transport and catabolism, and other minor pathways, significant differences were observed in all other subfunctions between the groups (Supplementary Table S12).

## 4. Discussion

Numerous studies have demonstrated that pollen affects the health of honeybees. After bees feed on pollen, they secrete royal jelly from the lower glands to feed the larvae. A pollen diet also reduces the larva development time in honeybees and increases pupal weight [31]. It also increases the resistance of honeybees to the external environment, maintains their health, and maintains their colony growth. The pollen diet, specifically its type, influences the health and gut microbiota of honeybees.

Different pollens vary in their contents of proteins, amino acids, lipids, and fatty acids. A slight difference was observed in the protein content of the three pollen types. However, lipid content in SBP was lower, amino acid content in the three pollen types was quite different, and essential amino acid content in SBP was the lowest. These results are a result of the relationship with the plant source, storage conditions, and seasons of pollen [32]. During feeding, the amount of SBP fed was the highest, whereas that of LBP was the lowest. The nutrient deposition of honeybees reflects the nutrient content of pollen. Fat deposition is lower in bees fed on SBP than in those fed on the other two pollen types. There is an important link between the protein and fat content of bees and the protein and fat content of their food. On the one hand, measuring the protein and fat content of bees can be a good indicator of the nutritional status of bees; on the other hand, the protein and fat content of honeybees is related to the longevity of bees and their resistance to disease [33]. Several explanations may be available for this result. First, this may be due to the variations in the feeding preferences of honeybees. For example, when the reward for pollen feed is the same, honeybees are more likely to collect apricot pollen than pear pollen [34]. Second, LBP has a high content of essential amino acids; worker honeybees that feed on LBP meet their nutritional needs in the early feeding. Therefore, they do not need to consume too much LBP to meet their own nutritional needs in the later stage. SBP is low in essential amino acids; consequently, honeybees that feed on SBP increase their feeding intake to fulfill their nutrient deficiencies. By contrast, honeybees may regulate their feed intake to prevent a pollen nutrient from exceeding its own threshold [35]. The LBP used in this experiment had high levels of trace elements such as sodium and potassium [36]. Because lipid content in SBP was lower, lipid deposition in the bodies of honeybees feeding on SBP was lower. Moreover, digestibility is a crucial factor affecting feed intake.

Food is digested and absorbed in the midgut, which is the most vital digestive organ of honeybees. This organ is also an integral part of the brain–gut axis and plays a major regulatory role in various processes in honeybees [37]. The pollen protein digestibility in honeybees is associated with the activity of digestive enzymes of the midgut [25]. It is also closely related to the shape of pollen particles and protein composition [38]. The study unveils that when the pollen grain fragmentation rate was calculated, the pollen digestibility of honeybees is 30–70%, and the nitrogen digestion rate, measured using the chromium trioxide labeling method, is 77–83% [39,40]. In our study, the pollen protein digestibility in honeybees was between 58% and 78%. Furthermore, the protein digestibility of SBP was the lowest, whereas that of LBP was the highest. This may be a result of differences in test methods. One additional possible explanation for our findings is the variation in pollen particle shapes or differences in protein composition, which may render SBP less suitable for digestion by honeybees. LBP has spherical pollen grains with three germination grooves and a surface with granular ornamentation formed by short basal columns, MBP is spherical with a single pore, indicating an inconspicuous ornamentation, and SBP is spherical with inconspicuous three-pore grooves and a surface with short spines. Sunflower belongs to the Asteraceae family, and Asteraceae pollen is characterized by low digestibility [41]. Another factor could be differing evaporation rates of water from the different pollen diets. Although we controlled environmental and beehive humidity before feeding and weighing, discrepancies in evaporation rates remain a potential confounding factor. Additionally, honeybees fed sunflower pollen exhibited the lowest intestinal lipase activity, likely due to its lower lipid content. In our study, these bees had thinner midgut walls than those consuming MBP and LBP, possibly due to differences in nutrient composition, which may also explain the lower digestibility observed with SBP.

Different pollen types also exert varied effects on the honeybee immune system. Studies have shown that pollen diets can modulate immune enzyme activity to counteract the microsporidian parasite *Nosema ceranae* infections, albeit with varying efficacy [9]. In our experiments, honeybees fed SBP exhibited elevated PO and Lys activities, along with increased expression of immunity-related genes (e.g., *ApoD*, *GstD1*, *Cyp4g11*, and *MKP3*). This may be attributed to the nutrient content of SBP, which may activate the host immune response. By contrast, MBP and LBP may not activate the immune response of the honeybees, but sunflower pollen activates the immune response of the host. Notably, these immune differences might diminish with the age of honeybees, and environmental contaminants could also contribute to immune responses, potentially causing irreversible changes that adversely affect pollinating insects, which are vital to ecosystem stability [42].

Pollen is rich in antioxidants, such as carotenoids, glutathione, phytoalexins, flavonoids, polyphenols, and vitamins C and E, which contribute to free radical scavenging and antioxidant activity [43]. Chestnut pollen, for instance, has demonstrated protective effects against DNA oxidation [44]. Enhanced activities of SOD, CAT, and GSH-Px have been observed in the tissues of rainbow trout (*Oncorhynchus mykiss*) fed chestnut pollen, with corresponding significant reductions in MDA content [45]. The activities of SOD, CAT, GST and GPx were increased in the hemolymph and fat bodies of pollen-fed worker honeybees compared to those fed sugar [46]. In our study, honeybees fed on SBP exhibited higher antioxidant enzyme activity, whereas those fed on LBP showed the highest total antioxidant capacity. The total antioxidant capacity appears to be closely related to the number of antioxidants present in pollen. Honeybees feeding on SBP had higher MDA levels, possibly because SBP contains certain strong oxidizing substances. Therefore, the relationship between pollen composition and antioxidant function must be further explored.

Determining the intestinal microbiota of honeybees can well elucidate the effects of different pollen on their health. Honeybee gut microbiota plays a critical role in host nutrition, weight gain, endocrine signaling, immune function, and pathogen resistance, and disruptions in the microbiota can compromise host health [47]. Many studies have shown that pollen impacts gut microbial composition, often improving microbial diversity to promote health. For example, the feeding of fresh pollen by nurse honeybees maintains the microbial health of the colony and reduces the prevalence of the pathogen *F. perrara* and the number of *Nosema* spp. spores [48]. Our findings showed that honeybees fed SBP had higher out counts. The gut microbiota composition of honeybees feeding SBP also differed significantly from those consuming MBP or LBP. This suggests that different pollens affect the abundance and composition of gut microbiota in honeybees. Notably, honeybees fed lotus pollen exhibited lower *Lactobacillaceae* abundance but higher *Acetobacteraceae* abundance, potentially explaining their enhanced protein digestibility. *Lactobacillaceae* produce various carbohydrate-metabolizing enzymes such as polysaccharide hydrolase and pectin degrading enzyme to promote carbohydrate metabolism, while *Acetobacteraceae* contribute to digestion and sugar metabolism [49]. Honeybees fed SBP had higher *Enterobacteriaceae* and *Bifidobacteriaceae* levels. *Bifidobacteriaceae* are critical for maintaining honeybee colony health and metabolism [50], particularly in adult workers, suggesting that SBP may accelerate the aging of honeybees. At the same time, honeybee immunity, increased antioxidant enzyme activity, and upregulation of immune gene expression in SBP also appeared to be related to these two microbiotas.

The intricate interplay between nutrition and gut microbiota complicates efforts to disentangle their respective impacts on honeybee health [51,52]. The type and form of nutrients consumed by honeybees determine the substrates available for microbial digestion, while metabolites produced by intestinal microorganisms also affect host health [12,53]. Dietary changes have been linked to variations in honeybee weight [54]. For example, feeding different pollens to honeybee larvae can affect the pupa weight. Changes in microbial composition or abundance affect honeybee growth, development, immunity, and resistance to pathogens [55,56,57]. While nutrition and gut microbiota likely have additive effects on honeybee’s health, further research is needed to elucidate these interactions.

## 5. Conclusions

Our research underscores the significant effects of pollen type on honeybee digestion, metabolism, overall health, immune function, and gut microbiota. Systematic analysis reveals that selecting appropriate pollen as feed can effectively enhance microbial diversity, stabilize gut microbial communities, and modulate health-related gene expression and enzyme activities. Future studies should further explore the multidimensional effects of diverse pollen diets on honeybee health and ecological contributions.

## Figures and Tables

**Table 1:** PCR primers used in this study.

Target Gene	Primer Sequences (5′–3′)	Description	GenBank Number
*β-Actin*	F: CCGTGATTTGACTGACTACCT	Standard control primer, forward	NM_001185145
	R: AGTTGCCATTTCCTGTTC	Standard control primer, forward	
*ApoD*	F: GAAGCGTGCGAGATTCTGTTCC	Standard control primer, forward	XM_392555.7
	R: CAGGTGATATTGCCGACGAGTTG	Standard control primer, forward	
*MKP3*	F: ATATCCCGAGTGGTGTGAAGGTAG	Standard control primer, forward	XM_006564514
	R: CTGGCTCCAATGATCGCTTATAGG	Standard control primer, forward	
*Cyp4g11*	F: CCGCCTGCCTTGCCGTTG	Standard control primer, forward	XM_006559340.3
	R: AGAAGACCATCGCCAAGCCATG	Standard control primer, forward	
*GstD1*	F: AAACCCGCAACACACGATACCC	Standard control primer, forward	NM_001178028.1
	R: CGATAGCGCCGTGCCGATG	Standard control primer, forward	

**Table 2:** Protein, Lipid, Carbohydrate, Ash, and Energy contents of pollen.

Content	Types of Bee Pollen		
	MBP	LBP	SBP
Protein (%)	15.14 ± 1.52	15.37 ± 1.39	16.69 ± 1.45
Lipid (%)	6.87 ± 0.19 ^a^	6.27 ± 0.51 ^a^	4.15 ± 0.32 ^b^
Carbohydrate (%)	75.44 ± 1.48	72.79 ± 1.72	76.86 ± 1.88
Crude ash (%)	2.54 ± 0.04 ^b^	5.57 ± 0.15 ^a^	2.30 ± 0.37 ^b^
Energy (Kcal/100 g)	424.19 ± 0.87 ^a^	409.08 ± 2.85 ^b^	411.57 ± 1.95 ^b^

MBP: maize bee-collected pollen, LBP: lotus bee-collected pollen, SBP: sunflower bee-collected pollen. The same below. Data are presented as mean ± SEM of three independent experiments (*n* = 5). Different letters above bars indicate significant differences between two groups (*p* < 0.05), as determined by one-way ANOVA with Tukey’s multiple comparison test.

**Table 3:** Amino acid contents of pollen.

Amino Acid (g/100 g)	Types of Bee Pollen		
	MBP	LBP	SBP
* Histidine	0.29 ± 0.03 ^c^	0.39 ± 0.02 ^b^	0.54 ± 0.05 ^a^
* Methionine	0.10 ± 0.02	0.08 ± 0.03	0.10 ± 0.05
* Threonine	0.64 ± 0.02 ^a^	0.64 ± 0.01 ^a^	0.52 ± 0.03 ^b^
* Valine	0.77 ± 0.03 ^a^	0.79 ± 0.01 ^a^	0.62 ± 0.03 ^b^
* Tyrosine	0.33 ± 0.02 ^a b^	0.36 ± 0.03 ^a^	0.31 ± 0.02 ^b^
* Lysine	0.84 ± 0.02 ^b^	0.94 ± 0.03 ^a^	0.63 ± 0.04 ^c^
* Isoleucine	0.61 ± 0.02 ^b^	0.69 ± 0.02 ^a^	0.55 ± 0.03 ^c^
* Leucine	0.93 ± 0.03 ^b^	1.06 ± 0.02 ^a^	0.88 ± 0.05 ^b^
* Phenylalanine	0.55 ± 0.02 ^b^	0.66 ± 0.02 ^a^	0.52 ± 0.04 ^b^
* Arginine	0.63 ± 0.02 ^b^	0.71 ± 0.01 ^a^	0.50 ± 0.02 ^c^
Serine	0.38 ± 0.01 ^a^	0.39 ± 0.01 ^a^	0.27 ± 0.02 ^b^
Glycine	0.68 ± 0.02	0.67 ± 0.01	0.67 ± 0.05
Aspartic acid	0.93 ± 0.01 ^b^	1.25 ± 0.03 ^a^	0.86 ± 0.09 ^b^
Glutamic acid	1.32 ± 0.02 ^b^	1.69 ± 0.02 ^a^	1.20 ± 0.08 ^c^
Alanine	0.92 ± 0.03 ^a^	0.81 ± 0.01 ^b^	0.67 ± 0.05 ^c^
Proline	2.15 ± 0.06 ^a^	0.59 ± 0.01 ^c^	0.81 ± 0.05 ^b^
Cystine	0.10 ± 0.01	0.10 ± 0.02	0.14 ± 0.04

Amino acid contents of three pollen types. “*” indicates essential amino acids. Data are presented as mean ± SEM of three independent experiments (*n* = 3). Different letters above the bars indicate significant differences between two groups (*p* < 0.05), as determined by one-way ANOVA with Tukey’s multiple comparison test.

## Data Availability

The original contributions presented in the study are included in the article, further inquiries can be directed to the corresponding author.

## Supplementary Materials

The following supporting information can be downloaded at: https://www.mdpi.com/article/10.3390/insects16050505/s1, Figure S1: The rarefaction analyses of all samples. Rarefaction curves generated from the OTUs suggested that high sampling coverage was achieved in all samples. Figure S2: Species OTU number Venn diagram. Table S1: The Elution gradient of High-performance liquid chromatography (HPLC). Table S2: Statistical results of data preprocessing. Table S3: Statistics of OTUs and tags of different samples. Table S4: Comparative results of the relative abundances of the five most abundant bacterial phyla for the hindgut samples. Table S5: Comparative results of the relative abundance of the five most abundant bacterial genera present in the hindgut samples. Table S6: Alpha diversity of bee hindgut samples studied. Table S7: Comparison of alpha-diversity indexes of samples based on Tukey’s HSD. Table S8: PERMANOVA statistical tests performed with unweighted UniFrac distances and Bray–Curtis dissimilarity for three bee pollen. Table S9: Indicator species analyses at the genus level of bee hindgut samples. Table S10: Pearson’s correlation analysis results between the nutritional components of pollen and the microbial genera in the honeybee gut. Table S11: Comparison of PICRUSt2-based microbial function prediction (levels 1 and 2) of different samples. Table S12: Comparison of PICRUSt2-based microbial function prediction (levels 1 and 2) of different samples.
