# Supplementary material for: Effects of Three Different Bee Pollen on Digestion, Immunity, Antioxidant Capacity, and Gut Microbes in Apis mellifera
Source: Insects. 2025 May 8;16(5):505. doi: 10.3390/insects16050505 (PMC12112133; doi:10.3390/insects16050505)
Supplement: Supplementary file 1 [file insects-16-00505-s001.zip › Figure S1 .pdf]

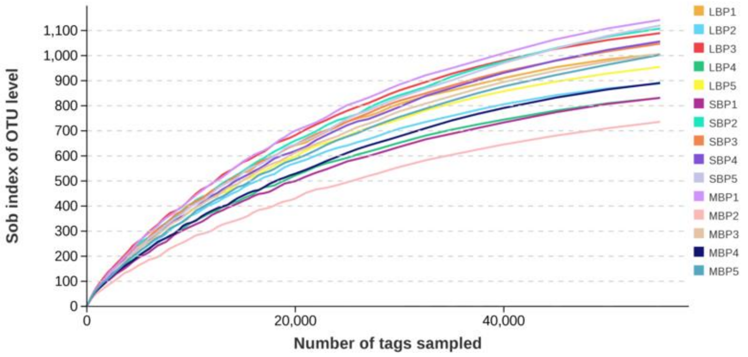

Figure S1. The rarefaction analyses of all samples. Rarefaction curves generated from the OTUs suggested that high sampling coverage was achieved in all samples. MBP (MBP-1, MBP-2, MBP-3, MBP-4, MBP-5): Maize bee pollen. **LBP (LBP-1, LBP-2, LBP-3, LBP-4, LBP-5)**: Lotus bee pollen. **SBP (SBP-1, SBP-2, SBP-3, SBP-4, SBP-5)**: Sunflower bee pollen.
