# Supplementary material for: Effects of Three Different Bee Pollen on Digestion, Immunity, Antioxidant Capacity, and Gut Microbes in Apis mellifera
Source: Insects. 2025 May 8;16(5):505. doi: 10.3390/insects16050505 (PMC12112133; doi:10.3390/insects16050505)
Supplement: Supplementary file 1 [file insects-16-00505-s001.zip › Figure S2.pdf]

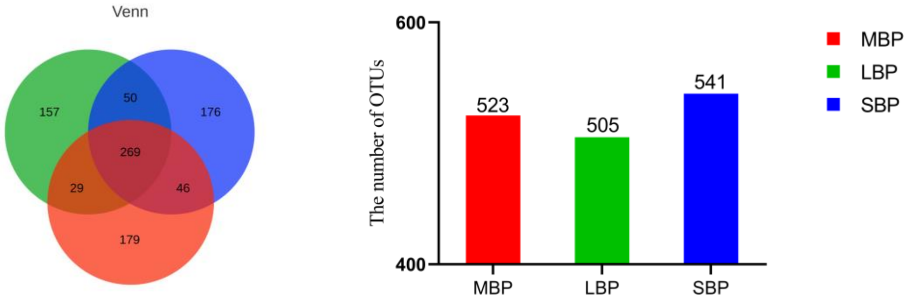

Figure S2. Species OTU number Venn diagram.MBP: Maize bee pollen. **LBP** : Lotus bee pollen. **SBP** : Sunflower bee pollen.
