# Supplementary material for: Effects of Three Different Bee Pollen on Digestion, Immunity, Antioxidant Capacity, and Gut Microbes in Apis mellifera
Source: Insects. 2025 May 8;16(5):505. doi: 10.3390/insects16050505 (PMC12112133; doi:10.3390/insects16050505)
Supplement: Supplementary file 1 [file insects-16-00505-s001.zip › Tables S1-S12.pdf]

Table S1. The Elution gradient of High performance liquid chromatography (HPLC).

Time（min）	Mobile phase	
	A（v%）	B（v%）
0	96	4
0.5	96	4
2.5	90	10
5	72	28
6	5	95
7	5	95
7.1	96	4
9	96	4

Table S2. Statistical results of data preprocessing. MBP (MBP-1, MBP-2, MBP-3, MBP-4, MBP-5): Maize bee pollen. **LBP (LBP-1, LBP-2, LBP-3, LBP-4, LBP-5)**: Lotus bee pollen. **SBP (SBP-1, SBP-2, SBP-3, SBP-4, SBP-5)**: Sunflower bee pollen.

Sample Name	Raw PE	Clean PE	Raw Tags	Clean Tags	Effective Tags	Effective Ratio (%)
MBP-1	135671	135630	134897	133630	103930	76.60
MBP-2	127397	127367	126742	126071	102876	80.75
MBP-3	122405	122365	121628	119863	96567	78.89
MBP-4	123198	123159	122384	122108	103138	83.72
MBP-5	126504	126467	125705	125391	104704	82.77
LBP-1	113783	113759	113191	112576	88337	77.64
LBP-2	120792	120759	120057	118520	91992	76.16
LBP-3	123956	123927	123269	122867	97153	78.38
LBP-4	129246	129206	128463	126233	97115	75.14
LBP-5	131130	131105	130378	128895	101031	77.05
SBP-1	132309	132284	131482	130609	101088	76.40
SBP-2	134571	134528	133755	133540	105934	78.72
SBP-3	127437	127401	126757	126450	98595	77.37
SBP-4	124451	124421	123552	123255	99291	79.78
SBP-5	137460	137425	136820	136670	108596	79.00

Table S3. Statistics of OTUs and tags of different samples. MBP (MBP-1, MBP-2, MBP-3, MBP-4, MBP-5): Maize bee pollen. **LBP (LBP-1, LBP-2, LBP-3, LBP-4, LBP-5)**: Lotus bee pollen. **SBP (SBP-1, SBP-2, SBP-3, SBP-4, SBP-5)**: Sunflower bee pollen.

Sample ID	Total Tags	Taxon Tags	Unclassified Tags	Singleton Tags	OTUs
MBP-1	103930	65967	0	37963	1193
MBP-2	102876	71147	0	31729	786
MBP-3	96567	67036	2	29529	1055
MBP-4	103138	71281	0	31857	957
MBP-5	104704	71702	0	33002	1075
LBP-1	88337	57449	0	30888	1014
LBP-2	91992	58260	2	33730	900
LBP-3	97153	61067	2	36084	1113
LBP-4	97115	60242	0	36873	851
LBP-5	101031	62711	0	38320	981
SBP-1	101088	67272	2	33814	880
SBP-2	105934	70937	0	34997	1184
SBP-3	98595	62879	0	35716	1080
SBP-4	99291	67470	0	31821	1113
SBP-5	108596	75063	0	33533	1218
Avg	100023	66032	0	33990	1026

Table S4. Comparative results of the relative abundances of the 5 most abundant bacterial phyla for the hindgut samples. MBP: Maize bee pollen. **LBP**: Lotus bee pollen. **SBP**: Sunflower bee pollen. Values are means ± SEM (n=5). Statistical analysis was performed by Tukey's HSD test, and different letters in the same column (a, b, c) indicate a significant difference at P<0.05.

**Groups**	**Proteobacteria**	**Firmicutes**	**Bacteroidetes**	**Actinobacteria**	**Acidobacteriota**
MBP	37.05±5.22b	55.19±4.49a	0.76±0.21a	3.59±0.85a	0.48±0.08a
LBP	52.40±1.57a	39.07±1.57b	0.77±0.07a	4.11±0.27a	0.55±0.09a
SBP	37.26±3.32b	52.90±3.04a	0.60±0.04a	5.50±0.35a	0.56±0.05a
F	5.701	7.169	0.546	3.168	0.344
P	0.018	0.009	0.593	0.079	0.715

Table S5. Comparative results of the relative abundance of the 5 most abundant bacterial genera present in the hindgut samples. MBP: Maize bee pollen**. LBP:** Lotus bee pollen**. SBP:** Sunflower bee pollen**.** *P<0.05, **P<0.01 and ***P<0.001, by Tukey HSD test.

**Sample**	***Lactobacillus***	***Frischella***	***Commensalibacter***	***Gilliamella***	***Bifidobacterium***
MBP-1	45.2969	8.3011	12.338	17.6118	1.8343
MBP-2	58.6392	7.9582	9.5211	0.4301	2.752
MBP-3	43.8468	5.5536	24.5622	17.1947	1.2769
MBP-4	60.4102	15.8219	1.0957	0.4966	5.2553
MBP-5	66.8126	9.5953	1.6415	0.4156	5.6581
LBP-1	42.6726	16.6565	14.3101	3.2115	3.8504
LBP-2	36.5384	15.0784	21.7964	9.8829	4.4231
LBP-3	34.9703	19.5877	12.0503	17.2019	3.1129
LBP-4	37.9138	11.5202	22.886	8.509	4.7176
LBP-5	42.538	5.5636	19.8625	6.8792	3.8111
SBP-1	48.9178	17.072	7.9971	9.4747	5.3245
SBP-2	47.7832	22.0999	0.7373	5.3935	5.3498
SBP-3	46.8837	14.5072	2.3951	11.4887	4.5325
SBP-4	59.3864	7.6464	0.7974	1.1101	4.9103
SBP-5	60.9261	5.6952	0.822	1.1017	6.5811

**Genus**	**Group 1**	**Group 2**	**P**	**P. signif**	**Method**
	MBP	LBP	0.011	*	Tukey HSD
Lactobacillus	MBP	SBP	0.881	ns	Tukey HSD
	LBP	SBP	0.027	*	Tukey HSD
	MBP	LBP	0.462	ns	Tukey HSD
Frischella	MBP	SBP	0.506	ns	Tukey HSD
	LBP	SBP	0.996	ns	Tukey HSD
	MBP	LBP	0.142	ns	Tukey HSD
Commensalibacter	MBP	SBP	0.214	ns	Tukey HSD
	LBP	SBP	0.006	**	Tukey HSD

	MBP	LBP	0.896	ns	Tukey HSD
Gilliamella	MBP	SBP	0.933	ns	Tukey HSD
	LBP	SBP	0.707	ns	Tukey HSD
	MBP	LBP	0.726	ns	Tukey HSD
Bifidobacterium	MBP	SBP	0.074	ns	Tukey HSD
	LBP	SBP	0.256	ns	Tukey HSD
	MBP	LBP	0.539	ns	Tukey HSD
Enterobacter	MBP	SBP	0.028	*	Tukey HSD
	LBP	SBP	0.179	ns	Tukey HSD

Table S6. Alpha diversity of bee hindgut samples studied. MBP (MBP-1, MBP-2, MBP-3, MBP-4, MBP-5): Maize bee pollen. **LBP (LBP-1, LBP-2, LBP-3, LBP-4, LBP-5)**: Lotus bee pollen. **SBP (SBP-1, SBP-2, SBP-3, SBP-4, SBP-5)**: Sunflower bee pollen.

**Sample**	**Sobs**	**Shannon**	**Chao1**	**Ace**	**Goods coverage**
MBP-1	1014	3.66	1234.41	1282.91	1.00
MBP-2	131	3.71	813.09	846.45	1.00
MBP-3	146	3.27	1101.63	1151.47	1.00
MBP-4	97	3.91	993.31	1041.37	1.00
MBP-5	123	3.93	1118.73	1169.87	1.00
LBP-1	1014	3.53	1047.43	1092.61	1.00
LBP-2	900	3.56	935.49	970.79	1.00
LBP-3	1113	3.54	1146.75	1193.45	1.00
LBP-4	851	3.49	898.66	953.73	1.00
LBP-5	981	3.91	1021.81	1070.11	1.00
SBP-1	880	3.63	932.16	987.82	1.00
SBP-2	1184	3.65	1222.86	1270.24	1.00
SBP-3	1080	3.85	1126.43	1177.61	1.00
SBP-4	1113	3.47	1166.35	1216.47	1.00
SBP-5	1218	3.20	1256.63	1312.07	1.00

Table S7. Comparison of alpha-diversity indexes of samples based on Tukey’s HSD test (n=5). MBP: Maize bee pollen. **LBP**: Lotus bee pollen. **SBP**: Sunflower bee pollen.

**Index**	**p value**			**Method**
	**MBP vs LBP**	**MBP vs SBP**	**LBP vs SBP**	Tukey HSD
**Sobs**	0.872	0.596	0.328	Tukey HSD
**Shannon**	0.815	0.632	0.947	Tukey HSD
**Chao1**	0.867	0.546	0.288	Tukey HSD
**Ace**	0.870	0.515	0.270	Tukey HSD

Table S8. PERMANOVA statistical tests performed with unweighted UniFrac distances and Bray-Curtis dissimilarity for three bee pollen.MBP: Maize bee pollen. **LBP**: Lotus bee pollen. **SBP**: Sunflower bee pollen. *P<0.05, and **P<0.01.

**Metric**	**Variable**	**F**	**R^2**	***P***	***P*. signif**
Unweighted UniFrac	MBP-vs-LBP	1.058	0.1168	0.038	*
	MBP-vs-SBP	1.0514	0.1162	0.046	*
	LBP-vs-SBP	1.036	0.1146	0.135	ns
	MBP-vs-LBP-vs-SBP	1.0485	0.1488	0.012	*
Bray-Curtis	MBP-vs-LBP	2.9115	0.2688	0.097	ns
	MBP-vs-SBP	3.696	0.316	0.006	**
	LBP-vs-SBP	4.4811	0.359	0.011	*
	MBP-vs-LBP-vs-SBP	3.5767	0.3735	0.002	**

Table S9. Indicator species analyses at the genus level of bee hindgut samples. MBP: Maize bee pollen. **LBP**: Lotus bee pollen. **SBP**: Sunflower bee pollen. *P<0.05, and **P<0.01.

**Index**	**indicator value**			**p value**
	**MBP**	**LBP**	**SBP**	
Commensalibacter	0.322	0.595	0.083	0.017
Bifidobacterium	0.265	0.314	0.421	0.046
Enterobacter	0.105	0.288	0.607	0.029
Bombella	0.812	0.109	0.079	0.044
Pontibacter	0.205	0.470	0.325	0.046
Massilia	0.157	0.608	0.235	0.009

Table S10. Pearson's correlation analysis results between the nutritional components of pollen and the microbial genera in the honeybee gut. MBP: Maize bee pollen. **LBP**: Lotus bee pollen. **SBP**: Sunflower bee pollen.

Items	Digestibility		Protein		Lipid		Moisture		Ash		Carbohydrates	
	correlation	p_value	correlation	p_value	correlation	p_value	correlation	p_value	correlation	p_value	correlation	p_value
Lactobacillus	-0.720	0.029	-0.169	0.664	-0.412	0.270	0.332	0.383	-0.720	0.029	0.694	0.038
Frischella	0.027	0.946	0.111	0.777	-0.055	0.889	0.429	0.249	-0.024	0.951	-0.077	0.844
Commensalibacter	0.784	0.012	0.068	0.862	0.538	0.136	-0.478	0.193	0.687	0.041	-0.650	0.058
Gilliamella	0.296	0.439	0.119	0.759	0.478	0.193	-0.512	0.159	0.096	0.806	-0.367	0.331
Bifidobacterium	-0.245957	0.524	-0.080	0.839	-0.596	0.090	0.630	0.069	0.017	0.964	0.345	0.363
Enterobacter	-0.718769	0.029	0.124	0.750	-0.900	0.001	0.220	0.570	-0.420	0.260	0.528	0.144
Snodgrassella	0.750	0.020	-0.371	0.326	0.712	0.031	-0.220	0.570	0.690	0.040	-0.398	0.288
Bombella	0.093	0.812	-0.383579	0.308	0.441	0.235	0.296	0.439	-0.205	0.597	0.185	0.634
Bartonella	0.279	0.468	-0.120	0.759	0.559	0.118	-0.444	0.231	0.284	0.459	-0.296	0.439
Raoultella	-0.264	0.492	-0.092	0.814	-0.585	0.098	-0.237	0.540	-0.219	0.572	0.375	0.320
RB41	-0.712	0.031	-0.180	0.642	-0.068	0.862	0.278	0.468	-0.544	0.130	0.511	0.159
Pseudomonas	0.688	0.041	-0.120	0.759	-0.016	0.967	-0.154	0.692	0.757	0.018	-0.299	0.434
Sphingobacterium	-0.374	0.321	0.378	0.316	-0.390	0.299	0.570	0.109	-0.120	0.759	-0.029	0.941
Pontibacter	0.599	0.088	0.215	0.578	0.011	0.978	-0.146	0.708	0.645	0.061	-0.502	0.168
Candidatus_Kuenenia	0.369	0.328	-0.273	0.477	0.225	0.561	-0.255	0.509	0.589	0.095	-0.197	0.612
Serratia	-0.526	0.145	-0.056	0.886	-0.515	0.156	0.475	0.196	-0.256	0.506	0.493	0.178
Sphingobacterium	0.776	0.014	-0.345	0.363	0.007	0.987	-0.070	0.859	0.682	0.043	-0.140	0.720
Nitrospira	-0.440	0.236	0.052	0.894	-0.564	0.114	0.412	0.271	-0.035	0.929	0.243	0.529
Massilia	0.670	0.048	0.221	0.568	0.085	0.828	-0.458	0.215	0.611	0.080	-0.553	0.123
Melissococcus	0.080	0.839	-0.390	0.299	0.396	0.292	0.322	0.398	-0.218	0.573	0.213	0.581

Table S11. Comparison of PICRUSt2-based microbial function prediction (levels 1 and 2) of different samples. MBP: Maize bee pollen. **LBP**: Lotus bee pollen. **SBP**: Sunflower bee pollen.

Level_1	Level_2	MBP	LBP	SBP
Metabolism	Carbohydrate metabolism	330354.87	227107.91	330354.87
Metabolism	Amino acid metabolism	189799.29	162608.23	176064.47
Metabolism	Metabolism of cofactors and vitamins	176364.71	152344.55	159126.54
Metabolism	Metabolism of other amino acids	169726.69	132222.83	156013.59
Metabolism	Lipid metabolism	135918.38	93961.29	116512.66
Metabolism	Metabolism of terpenoids and polyketides	139535.18	97159.25	99215.85
Metabolism	Energy metabolism	102436.24	77652.62	85965.06
Metabolism	Xenobiotics biodegradation and metabolism	82730.43	67140.33	78962.61
Metabolism	Glycan biosynthesis and metabolism	67200.43	54684.72	60365.19
Metabolism	Nucleotide metabolism	47534.56	35767.51	45038.57
Metabolism	Biosynthesis of other secondary metabolites	24013.75	22927.15	20468.82
Genetic Information Processing	Replication and repair	153287.86	113621.33	141581.39
Genetic Information Processing	Translation	83718.04	62545.91	77653.670
Genetic Information Processing	Folding, sorting and degradation	71986.43	57778.55	64885.97
Genetic Information Processing	Transcription	14860.38	11364.75	13699.07
Environmental Information Processing	Membrane transport	99655.59	62535.64	78377.21
Environmental Information Processing	Signal transduction	8484.99	6486.86	7380.98
Environmental Information Processing	Signaling molecules and interaction	0.01	0.02	0.05
Cellular Processes	Cell growth and death	36265.42	28629.28	32349.75

Cellular Processes	Cell motility	18654.42	22532.81	13569.49
Cellular Processes	Transport and catabolism	3302.89	2998.06	3279.44
Cellular Processes	Cellular community - prokaryotes	3214.28	2633.39	3405.75
Human Diseases	Infectious diseases	5716.66	3867.98	5438.13
Human Diseases	Neurodegenerative diseases	1024.76	1177.60	536.46
Human Diseases	Cardiovascular diseases	4.27	7.24	6.52
Human Diseases	Immune diseases	3.13	4.98	4.83
Organismal Systems	Environmental adaptation	4388.79	3111.39	3377.30
Organismal Systems	Endocrine system	187.33	181.73	230.15
Organismal Systems	Immune system	216.75	168.55	146.19
Organismal Systems	Digestive system	160.59	148.26	201.80
Organismal Systems	Excretory system	0.07	0.21	0.18
Organismal Systems	Nervous system	0.02	0	0.03
Organismal Systems	Development	0	0.05	0

Table S12. Comparison of PICRUSt2-based microbial function prediction (levels 1 and 2) of different samples. MBP: Maize bee pollen. **LBP**: Lotus bee pollen. **SBP**: Sunflower bee pollen. by Tukey HSD test.

**Level_1**	**Level_2**	**p value**			**Method**
		**MBP vs LBP**	**MBP vs SBP**	**LBP vs SBP**	Tukey HSD
Metabolism	Carbohydrate metabolism	0.002	0.071	0.180	Tukey HSD
Metabolism	Amino acid metabolism	0.006	0.170	0.181	Tukey HSD
Metabolism	Metabolism of cofactors and vitamins	0.006	0.043	0.541	Tukey HSD
Metabolism	Metabolism of other amino acids	0.002	0.259	0.034	Tukey HSD
Metabolism	Lipid metabolism	0.003	0.173	0.104	Tukey HSD
Metabolism	Metabolism of terpenoids and polyketides	0.008	0.010	0.982	Tukey HSD
Metabolism	Energy metabolism	0.003	0.036	0.352	Tukey HSD
Metabolism	Xenobiotics biodegradation and metabolism	0.005	0.619	0.028	Tukey HSD
Metabolism	Glycan biosynthesis and metabolism	0.002	0.075	0.148	Tukey HSD
Metabolism	Nucleotide metabolism	0.003	0.646	0.014	Tukey HSD
Metabolism	Biosynthesis of other secondary metabolites	0.535	0.010	0.070	Tukey HSD
Genetic Information Processing	Replication and repair	0.003	0.455	0.030	Tukey HSD
Genetic Information Processing	Translation	0.004	0.488	0.031	Tukey HSD
Genetic Information Processing	Folding, sorting and degradation	0.001	0.064	0.063	Tukey HSD
Genetic Information Processing	Transcription	0.004	0.381	0.041	Tukey HSD
Environmental Information Processing	Membrane transport	0.003	0.073	0.204	Tukey HSD
Environmental Information Processing	Signal transduction	0.006	0.123	0.234	Tukey HSD
Environmental Information Processing	Signaling molecules and interaction	0.910	0.427	0.666	Tukey HSD
Cellular Processes	Cell growth and death	0.001	0.078	0.096	Tukey HSD
Cellular Processes	Cell motility	0.351	0.183	0.015	Tukey HSD

Cellular Processes	Transport and catabolism	0.228	0.990	0.277	Tukey HSD
Cellular Processes	Cellular community - prokaryotes	0.007	0.445	0.001	Tukey HSD
Human Diseases	Infectious diseases	0.003	0.795	0.008	Tukey HSD
Human Diseases	Neurodegenerative diseases	0.951	0.608	0.435	Tukey HSD
Human Diseases	Cardiovascular diseases	0.068	0.184	0.821	Tukey HSD
Human Diseases	Immune diseases	0.057	0.082	0.976	Tukey HSD
Organismal Systems	Environmental adaptation	0.001	0.003	0.530	Tukey HSD
Organismal Systems	Endocrine system	0.980	0.350	0.269	Tukey HSD
Organismal Systems	Immune system	0.928	0.854	0.984	Tukey HSD
Organismal Systems	Digestive system	0.896	0.327	0.169	Tukey HSD
Organismal Systems	Excretory system	0.196	0.357	0.909	Tukey HSD
Organismal Systems	Nervous system	0.761	0.956	0.593	Tukey HSD
Organismal Systems	Development	0.462	1	0.462	Tukey HSD
